# Enhanced Information Flow From Cerebellum to Secondary Visual Cortices Leads to Better Surgery Outcome in Degenerative Cervical Myelopathy Patients: A Stochastic Dynamic Causal Modeling Study With Functional Magnetic Resonance Imaging

**DOI:** 10.3389/fnhum.2021.632829

**Published:** 2021-06-23

**Authors:** Rui Zhao, Yingchao Song, Xing Guo, Xiaotian Yang, Haoran Sun, Xukang Chen, Meng Liang, Yuan Xue

**Affiliations:** ^1^Department of Orthopedics Surgery, Tianjin Medical University General Hospital, Tianjin, China; ^2^School of Medical Imaging, Tianjin Medical University, Tianjin, China; ^3^Department of Radiology, Tianjin Medical University General Hospital, Tianjin, China; ^4^Tianjin Key Laboratory of Spine and Spinal Cord, Tianjin Medical University General Hospital, Tianjin, China

**Keywords:** visual system, effective connectivity, dynamic causal model, fMRI, cerebellum, degenerative cervical myelopathy

## Abstract

Degenerative cervical myelopathy (DCM) damages the spinal cord, resulting in long-term neurological impairment including motor and visual deficits. Given that visual feedback is crucial in guiding movements, the visual disorder may be a cause of motor deficits in patients with DCM. It has been shown that increased functional connectivity between secondary visual cortices and cerebellum, which are functionally related to the visually guided movements, was correlated with motor function in patients with DCM. One possible explanation is that the information integration between these regions was increased to compensate for impaired visual acuity in patients with DCM and resulted in better visual feedback during motor function. However, direct evidence supporting this hypothesis is lacking. To test this hypothesis and explore in more detail the information flow within the “visual-cerebellum” system, we measured the effective connectivity (EC) among the “visual-cerebellum” system *via* dynamic causal modeling and then tested the relationship between the EC and visual ability in patients with DCM. Furthermore, the multivariate pattern analysis was performed to detect the relationship between the pattern of EC and motor function in patients with DCM. We found (1) significant increases of the bidirectional connections between bilateral secondary visual cortices and cerebellum were observed in patients with DCM; (2) the increased self-connection of the cerebellum was positively correlated with the impaired visual acuity in patients; (3) the amplitude of effectivity from the cerebellum to secondary visual cortices was positively correlated with better visual recovery following spinal cord decompression surgery; and (4) the pattern of EC among the visual-cerebellum system could be used to predict the pre-operative motor function. In conclusion, this study provided direct evidence that the increased information integration within the “visual-cerebellum” system compensated for visual impairments, which might have importance for sustaining better motor function in patients with DCM.

## Introduction

In clinical practice, degenerative cervical myelopathy (DCM) is a common disease, which results in compression of the cervical spinal cord, leading to numerous neurological dysfunctions, including sensory dysfunctions, fine motor deficits, and incontinence (Lebl et al., 2011; Akter and Kotter, 2018; Davies et al., 2018; Badhiwala et al., 2020). In recent years, several atypical symptoms including blurred vision, dizziness, headache, and tinnitus were also frequently reported (Sun et al., 2013, 2016; Muheremu et al., 2016). Among these neurological symptoms, the loss of fine motor control of the hands is the most frequently encountered symptom (Kalsi-Ryan et al., 2013), causing difficulty in the daily life of patients. At present, many researchers believe that sensorimotor disorders of DCM were due to the compression of the cord, eventually leading to disruption of neural transmission at the spinal level. However, emerging evidence suggests that cortical alterations may also be crucial in DCM pathology. At the brain level, it has been shown that the functional alterations within sensorimotor cortices occurred and associated with the motor deficits in patients with DCM (Tan et al., 2015; Liu et al., 2016); besides, these alterations show plasticity after decompression surgery (Zhou et al., 2015; Bhagavatula et al., 2016; Aleksanderek et al., 2017). Moreover, the functional alterations were not restricted to the sensorimotor cortices. Our earlier study showed that the regional neural activities within visual cortices were also altered and associated with the visual deficits in patients with DCM (Chen et al., 2018). Given that the visual feedback is crucial in the control of human movement (Sober and Sabes, 2005; Sarlegna et al., 2009; Goodale, 2011; Mizelle et al., 2016), visual impairments could be a crucial factor leading to motor deficits.

To probe the above issue, our previous study conducted seed-based functional connectivity (FC) analysis to explore the functional alterations of visual cortices in patients with DCM. We found that the FC between the dorsal visual stream regions [Brodmann Areas 7 (BA 7)] and the primary or secondary visual cortices (BA17/19) increased and associated with the impaired visual acuity in patients. More importantly, we found that the connection between secondary visual cortices [located in lateral occipital cortices (LOC)] and cerebellum was also increased and correlated with the impaired motor function in patients. Considering the importance of cerebellum and LOC in visual-motion perception, these findings indicated that abnormal visual processing may play an important role in motor deficits in patients with DCM. One possible explanation is that the information integration between these two regions was increased to compensate for impaired visual acuity in patients with DCM and resulted in better visual feedback during motor function (Chen et al., 2019).

Despite the acknowledged importance of LOC and cerebellum on visual perception (Perry and Fallah, 2014; Zachariou et al., 2014; Cooper and O'Sullivan, 2016; Ludwig et al., 2016; Galletti and Fattori, 2018), direct evidence supporting this hypothesis is still lacking. Besides, the previous study was conducted on FC analyses, while FC is based on temporal correlation between time courses extracted from two distinct brain regions (Pearson correlation), and it measures only the synchrony of undirectional neural activities. In contrast, effective connectivity (EC) measures the directional connection among brain regions and depicts the neural signals within one region exerted to those in another region. The EC analysis in DCM is thus particularly important for providing more mechanistic interpretations of the neuropathology of DCM. Therefore, to test our former hypothesis and explore in more detail the information flow within the “visual-cerebellum” system, we first estimated the EC among the primary visual cortex, bilateral visual cortices, and cerebellum, using dynamic causal modeling, which is one of the most popular and theoretically advanced methods for EC analysis, of resting-state fMRI data and then tested the relationship between the EC and visual ability in patients with DCM. Finally, multivariate pattern analysis (MVPA) was performed *via* support vector regression (SVR), a machine learning algorithm, to detect the relationship between the EC pattern of the “visual-cerebellum” system and motor function in patients with DCM.

## Materials and Methods

### Subjects

This prospective study was approved by the local Institutional Review Board of Tianjin Medical University General Hospital (Tianjin, China). The informed written consent was obtained from all participants before each procedure. A total of 27 right-handed patients with DCM were included for further analysis. The inclusion criteria were as follows: (a) clear evidence of cord compression on cervical spine MRI; (b) explicit clinical manifestations of sensorimotor extremities deficits or bladder and bowel dysfunction; (c) the patients agreed to undergo decompression of spinal canal; (d) no history of cervical spinal surgery; (e) able to complete the functional MRI (fMRI) studies; (f) no stenosis of extracranial vertebral artery and the carotid artery after Doppler ultrasound examination; (g) no clinical evidence or history of other neurological, psychiatric, ocular disease, or systemic disease, including hypertension and diabetes after consulting specialists from neurology, cardiology, and ophthalmology; and (h) no history of alcohol and substance abuse. Eleven healthy subjects of similar age, gender, and education (in years) were also recruited through advertisements with the following inclusion criteria: (a) no evidence of spinal compression, (b) no ocular disease, (c) no other spinal or brain neurological disorders, or systemic disease, and (d) able to complete the fMRI studies. The detailed information of our participants can be found in the study by Chen et al. (2018).

### Magnetic Resonance Data Acquisition and Pre-processing

All magnetic resonance (MR) data were acquired using a 3.0-T magnetic resonance scanner (Discovery MR750, General Electric) with a 32-channel phased-array head coil. Before scanning, the earplugs were placed inside the ears of the subjects to keep out noise, and the subjects were instructed to keep their head still during the scanning with a sponge pad to fix the head of the patient in order to minimize their unconscious activity and to keep their eyes closed but remain awake without specific and strong ideological activities. The functional images were collected using a gradient echo-planar pulse imaging sequence with the following parameters: repetition time, 2,000 ms; echo time, 30 ms; flip angle, 90°; field of view, 240 × 240 mm; matrix, 64 × 64; 38 slices; and slice thickness, 3.0 mm. The structural images were collected using a three-dimensional T1-weighted image (3D T1WI) for co-registration and normalization of the functional images, with the parameters being set as follows: sagittal acquisition, repetition time, 7.8 ms; echo time, 3.0 ms; inversion time, 450 ms; flip angle, 13°; field of view, 256 × 256 mm; matrix, 256 × 256; 180 slices; slice thickness, 1.0 mm. All subjects enrolled in this study underwent spinal cord decompression surgery after the fMRI scan.

The MR data were pre-processed with the toolbox Data Processing Assistant for rs-fMRI (DPARSF; http://www.restfmri.net/forum/DPARSF), and totally 180 volumes were acquired for a functional scan. The first 10 volumes of each functional scan were excluded for subjects adapting the scanning environment and magnetization stabilization. Slice-timing correction and motion correction were performed to remove timing differences and head movement, respectively. The functional images were co-registered with the structural images and spatially normalized to the Montreal Neurological Institute template, and each voxel was resampled to 3 × 3 × 3 mm^3^. Subsequently, the resampled images were smoothed with a 4-mm full-width half-maximum isotropic Gaussian kernel, linear trend and band-pass filter (0–0.08 Hz) was then applied to remove the effects of high-frequency noise. Finally, the six motion parameters, mean global signal, white matter signal, and CSF signal were extracted as covariates to reduce the non-neural signal, and the resulting data were used for further analysis.

### Clinical Assessment

The Japanese Orthopedic Association (JOA) scale, which is a commonly used scale in clinical practice to evaluate motor function in patients with DCM, was applied pre-operatively and at 6 months post-operatively for clinical evaluation. For visual acuity assessment, the Early Treatment in Diabetic Retinopathy Study (ETDRS) chart was used to evaluate the best-corrected visual acuity (BCVA, i.e., unaided, with appropriate spectacle or pinhole correction). The procedures have been discussed in detail in our published work (Chen et al., 2018). The brief procedures were as follows: all subjects were examined in the same room with standard distance (4 m) and light conditions. If a subject failed to read more than 20 letters at 4 m, then the BCVA was tested at 1 m. The ETDRS letter score was calculated as the number of letters recognized at 4 m plus 30 (or letters read at 1 m). We measured the left eye first followed by the right. All of these measurements were performed by an experienced clinical ophthalmologist.

To further test the relationship among these clinical measures, the correlation analyses were performed to assess the correlation among pre-/post-operative JOA scores, pre-/post-operative BCVA (both oculus dexter and oculus sinister), JOA recovery, and BCVA recovery (both oculus dexter and oculus sinister).

### Dynamic Causal Modeling

Dynamic causal modeling is an EC analysis method for making inferences about causal relationship between neural processes of different brain regions, which underlie measured BOLD fMRI time series (Friston et al., 2003). The general idea of Dynamic Causal Model is to construct a reasonably realistic neuronal system model and to infer corresponding parameters, so that the predicted BOLD time series corresponds as closely as possible to the observed BOLD signal. In this study, we adopted stochastic dynamic causal model (Li et al., 2011), and its particular advantage over the conventional Dynamic Causal Model is that it allows for modeling endogenous or random fluctuations in hidden neuronal and physiological states, enabling the analysis of resting state fMRI data.

**Figure 1:**
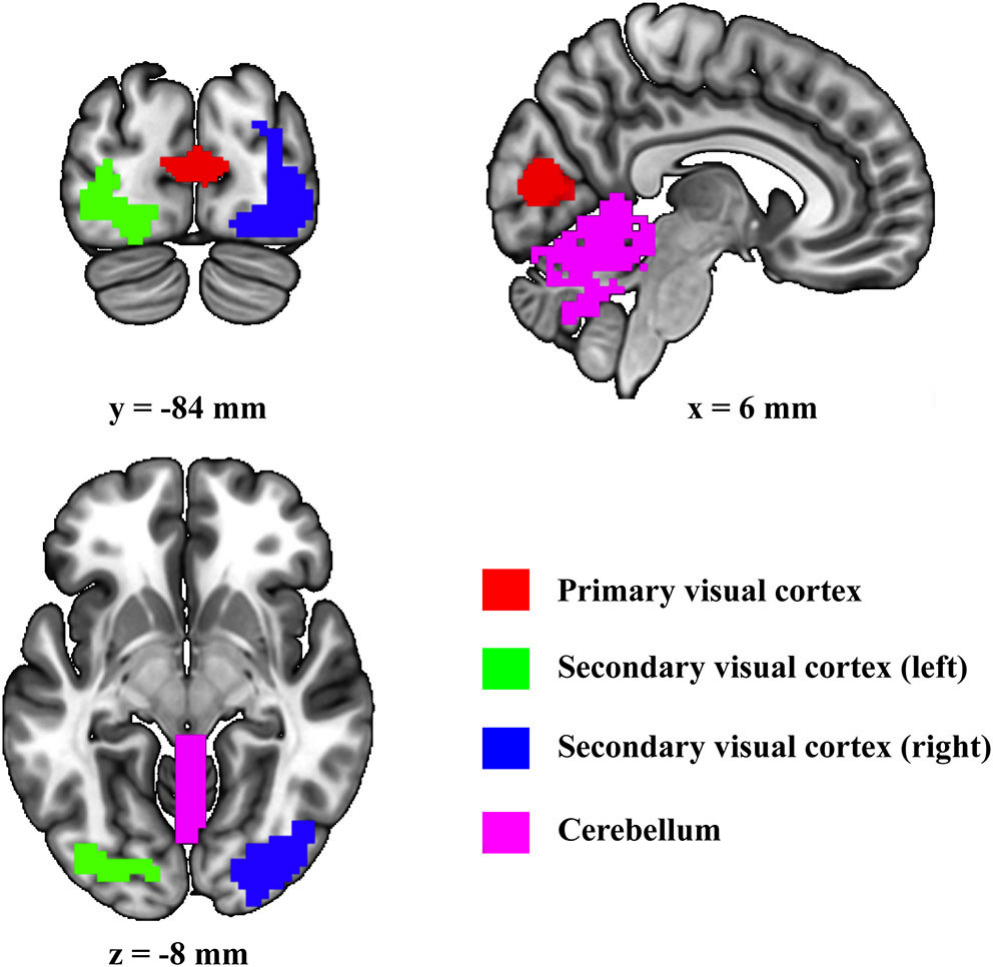
Location of the selected regions of interest.

First, the four regions of interest (ROIs) (i.e., cerebellum, primary visual cortex, left secondary visual cortex, and right secondary visual cortex) are included in present DCM (Figure 1). Primary visual cortex, left secondary visual cortex, and right secondary visual cortex were defined by using the template suggested by Shirer et al. (2012). Since it has been shown that the vermis is the only area related to visual processes in the cerebellum (Baumann et al., 2015), the cerebellum ROI was defined by using the Anatomical Automatic Labeling template by combining all the vermis subregions.

For each subject and each ROI, the fMRI time series were extracted by calculating the first eigen variate from all voxels included in the corresponding ROI. Second, a full Dynamic Causal Model with four ROIs was specified for each subject as implemented in (Statistical Parametric Mapping, http://www.fil.ion.ucl.ac.uk/spm). In a full Dynamic Causal Model, all possible connectivity parameters, that is, all bidirectional connections among the four ROIs and the self-connection of each ROI (cf., SPM procedure: *spm_dcm_fit*), were estimated. The exact options when constructing the full Dynamic Causal Model are as follows: non-linear model, one state per region, stochastic effects, and no centering. Then the full Dynamic Causal Model models were estimated for each subject. Finally, we conducted a group-level analysis for both the Dynamic Causal Model group and healthy controls, respectively, using the Parametric Empirical Bayesian (PEB) scheme in SPM12 (cf., SPM procedure: *spm_dcm_peb*) (Friston et al., 2015, 2016; Zeidman et al., 2019b). In brief, in PEB, the effective connection parameters averaged across all subjects in each group were estimated, taking into account the within-subject variability on the parameters. Once the group-level PEB model with a full connectivity configuration was obtained, the Bayesian Model Reduction (BMR) was used to prune away any insignificant connectivity parameters from the full connectivity model until the model evidence was not improved (cf., SPM procedure: *spm_dcm_peb_bmc*) (see Zeidman et al., 2019b for details). In this study, the connectivity parameters were considered significant when their posterior probability was *p* > 0.95. The two-sample *t*-tests were further performed on each connectivity parameter between the DCM group and healthy controls to test whether there is a significant difference between the two groups. Subsequently, we tested the correlations between the pre-PCVA values and the EC with a significant group difference.

### Multivariate Pattern Analysis

An MVPA was performed to detect the potential relationship between the EC pattern of the visual-cerebellum system and the JOA scale. As the input of the SVR model, all the EC parameters estimated in DCM analysis (see the “Dynamic Causal Modeling” section) were fed into the model as features. In order to ensure that derived models would generalize to new individuals, leave-one-out-cross-validation (LOOCV) was applied, and by each time, 1-fold of the samples act as the testing data for the regression model, and the remaining folds of the samples were used to train the model. To achieve better model performance, we adopted feature selection procedure as follows: for each cross-validation step, all features first ranked by the weights after the model was trained, the top 5% features with the highest weights were then selected and used to train a new model using the training dataset, and the newly trained model was tested using the test dataset. Subsequently, after all the cross-validation steps were finished, a correlation coefficient was calculated between the predict labels and the true labels. This feature selection procedure was repeated for a series of selected features from 5 to 100% with a step of 5% increment, resulting in 20 selected feature sets and thus 20 correlation coefficients. The statistical significance of the regression model was determined by the permutation test (*n* = 1,000) and corrected for multiple comparisons [*P* < 0.05, corrected for family-wise error (FWE)] using an in-house MATLAB (R2017a) script. In brief, in each permutation step, after randomly shuffling the labels of all samples, the SVR combined with feature selection were performed to generate corresponding *r* values, and the maximal *r* value across all models was selected; this procedure was repeated 1,000 times and resulted in 1,000 maximal absolute *r* values, which were used to generate the null distribution for calculating the *P*-value of each model. It is noted that, because the null distribution was generated using the maximal *r* values across all the SVR models, the resultant *P*-values were automatically corrected for FWE (Nichols and Hayasaka, 2003). All analyses were conducted in LIBSVM (www.csie.ntu.edu.tw/~cjlin/libsvm), using a linear kernel. The parameters of the SVM were set to its default value.

### Validation Analyses

The ROIs we selected in this study were all located at the posterior fossa. Possible distortions and susceptibility artifacts could be caused by the skull base. Therefore, we calculated the temporal signal-to-noise ratio (tSNR) [i.e., defined by the ratio of the SD of signal and the SD of noise (Calhoun et al., 2005; Penny, 2012)] of these ROIs and several contrast brain regions (e.g., precentral gyrus, middle frontal gyrus, insular, precuneus, and thalamus) and compared the tSNR between patients with DCM and healthy controls.

Moreover, we also used the Shirer's template to define the cerebellum ROI and performed all the above analyses to test the consistency of our results, and the ROIs used in this validation analyses are shown in Supplementary Figure 1.

We tested that the changes of EC within the “visual-cerebellum” system were specifically related to the spinal cord compression rather than the results of overall improvement in the patients. We also performed all analyses in the “Dynamic Causal Modeling” section using the ROIs within the auditory system. The ROIs used in these analyses are shown in Supplementary Figure 7.

## Results

### Demographic Data

The demographic data and behavior scale scores of this study and control groups are illustrated in Table 1. No significant difference between groups was observed in terms of age, gender, or education (in years) at *P* < 0.05. We found a moderate positive correlation between pre-operative visual acuity and JOA scores (*R* = 0.36, *P* < 0.05, uncorrected for OD; *R* = 0.35, *P* < 0.05, uncorrected for OS), between visual acuity recovery and JOA recovery (*R* = 0.38, *P* < 0.05, uncorrected for OS) (the detailed correlation coefficients among clinical measures were shown in Supplementary Table 1).

**Table 1:** The demographic data and clinical assessments of DCM patients and healthy controls.

**Characteristics**	**DCM (*n* = 27)**	**Healthy control (*n* = 11)**	***P*-value**
Age (years)	57.9 ± 9.1	54.8 ± 8.4	0.34
Gender (female/male)	12/15	5/6	0.96
Education (years)	10.8 ± 2.7	11.6 ± 2.5	0.42
JOA scale	11.8 ± 1.5		
Pre_OD	75.9 ± 3.8		
Pre_OS	76.1 ± 3.2		
Post_OD	78.2 ± 3.1		
Post_OS	77.8 ± 3.8		
Post_OD-Pre_OD	2.10 ± 3.1		
Post_OS-Pre_OS	2.12 ± 3.2		

*DCM, degenerative cervical myelopathy; Pre, pre-operative; Post, post-operative; OD, oculus dexter; OS, oculus sinister; JOA, Japanese Orthopedic Association*.

### Dynamic Causal Modeling

**Figure 2:**
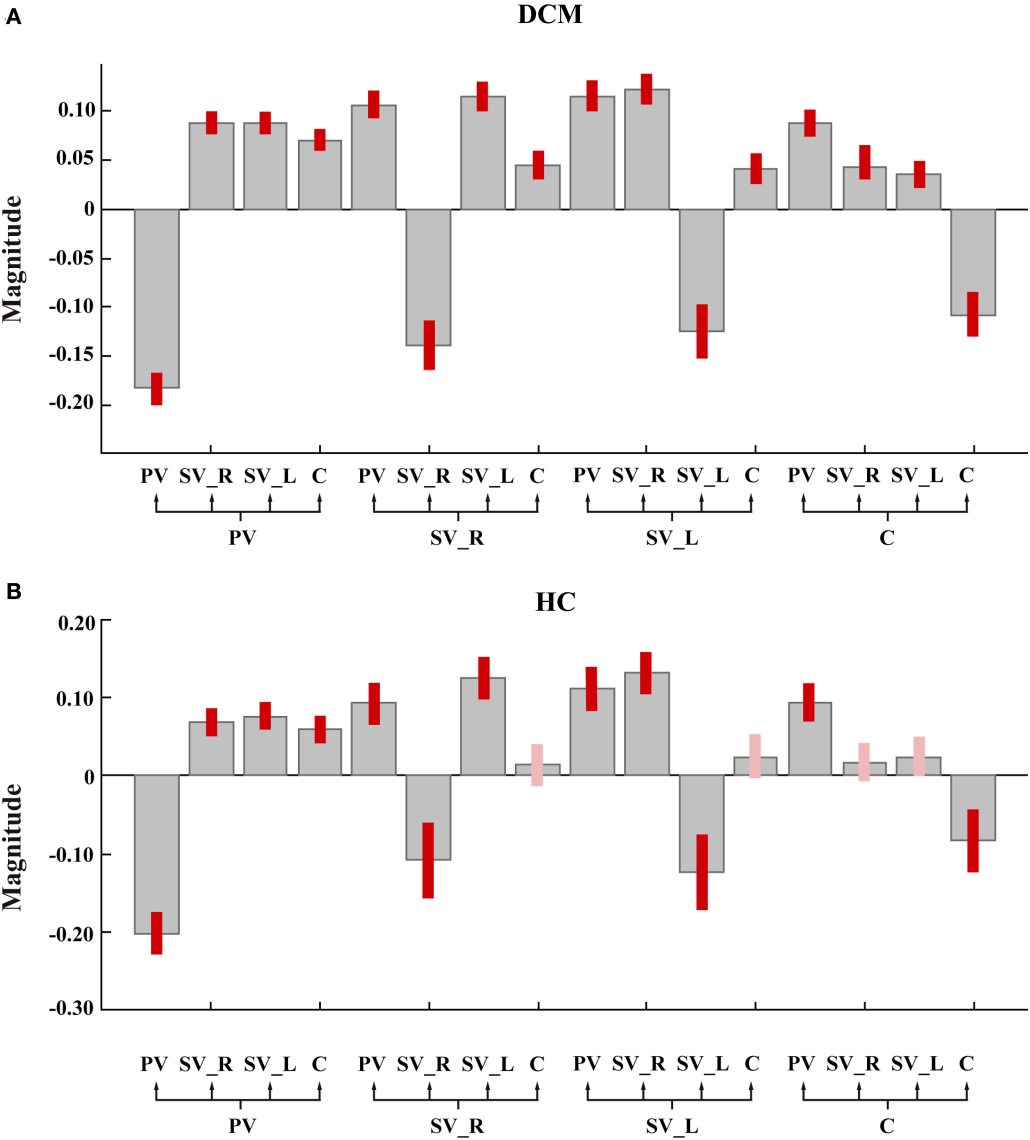
The group mean estimated intrinsic parameters. Gray-colored bars represent posterior means and pink-colored bars (or red-colored bars) represent 95% Bayesian confidence intervals. The red-colored bars represent the parameter whose posterior probability >0.95. PV, primary visual cortex; SV_L, left secondary visual cortex; SV_R, right secondary visual cortex; C, cerebellum. **(A)** The group mean estimated intrinsic parameters of degeneration cervical myelopathy patients. **(B)** The group mean estimated intrinsic parameters of healthy controls.

**Figure 3:**
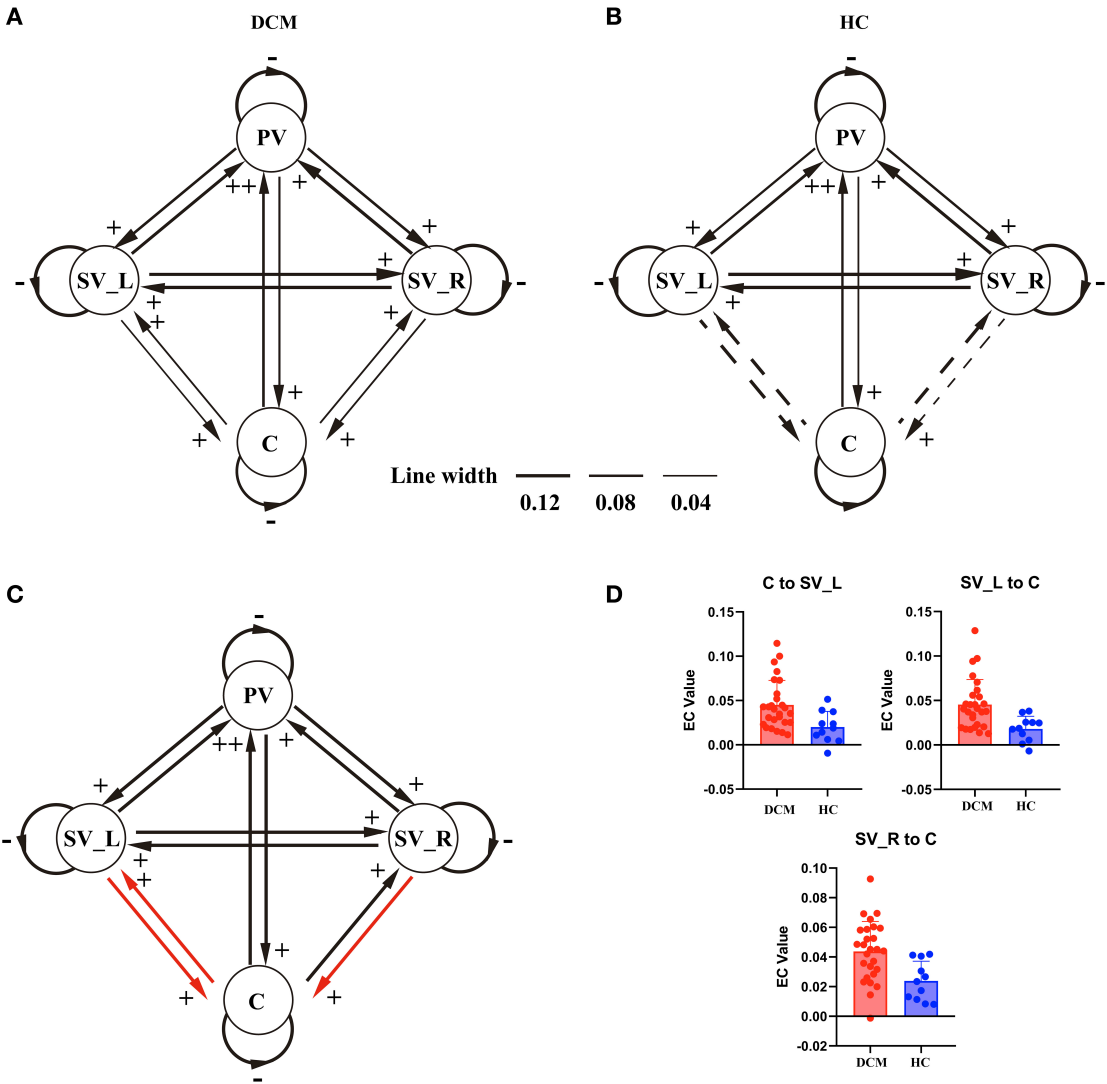
The hierarchical difference of visual-cerebellum network between groups. For **(A,B)**, final model structure determined by PEB and BMR. Black lines with arrowhead represent the intrinsic connections in the network and the thickness of the lines represent the magnitude of each connection (the solid lines represent significant intrinsic connections and the dotted lines represent insignificant intrinsic connections). Signs beside the arrowhead indicate the sign of the intrinsic connection. **(C)** Illustrated the model differences between patients and healthy controls. The red lines represent the connections showing significant differences between groups. **(D)** Showed the violin plots of the connections showing significant difference between groups. PV, primary visual cortex; SV_L, left secondary visual cortex; SV_R, right secondary visual cortex; C, cerebellum.

As shown in Figures 2A, 3A, in the final model structure of the DCM group, all bidirectional intrinsic connections among the cerebellum, primary visual cortex, left secondary visual cortex, and right secondary visual cortex were significantly positive; the self-connection of each ROI was significantly negative. As shown in Figures 2B, 3B, in the final model structure of healthy controls, all bidirectional intrinsic connections among the primary visual cortex, left secondary visual cortex, and right secondary visual cortex were significantly positive; the self-connections of the four ROIs were significantly negative. Similar to the cerebellum, only the bidirectional connections between the cerebellum and primary visual cortex were significantly positive, and other cerebellum-related connections, that is, the bidirectional connections between the cerebellum and left secondary visual cortex and the bidirectional connections between the cerebellum and right secondary visual cortex, were all insignificant.

The two-sample *t*-test results further confirmed that the strength of the bidirectional connections between the cerebellum and left secondary visual cortex and unidirectional connection from the right secondary visual cortex to cerebellum was significantly stronger in the DCM group than in control subjects (Figures 3C,D).

**Figure 4:**
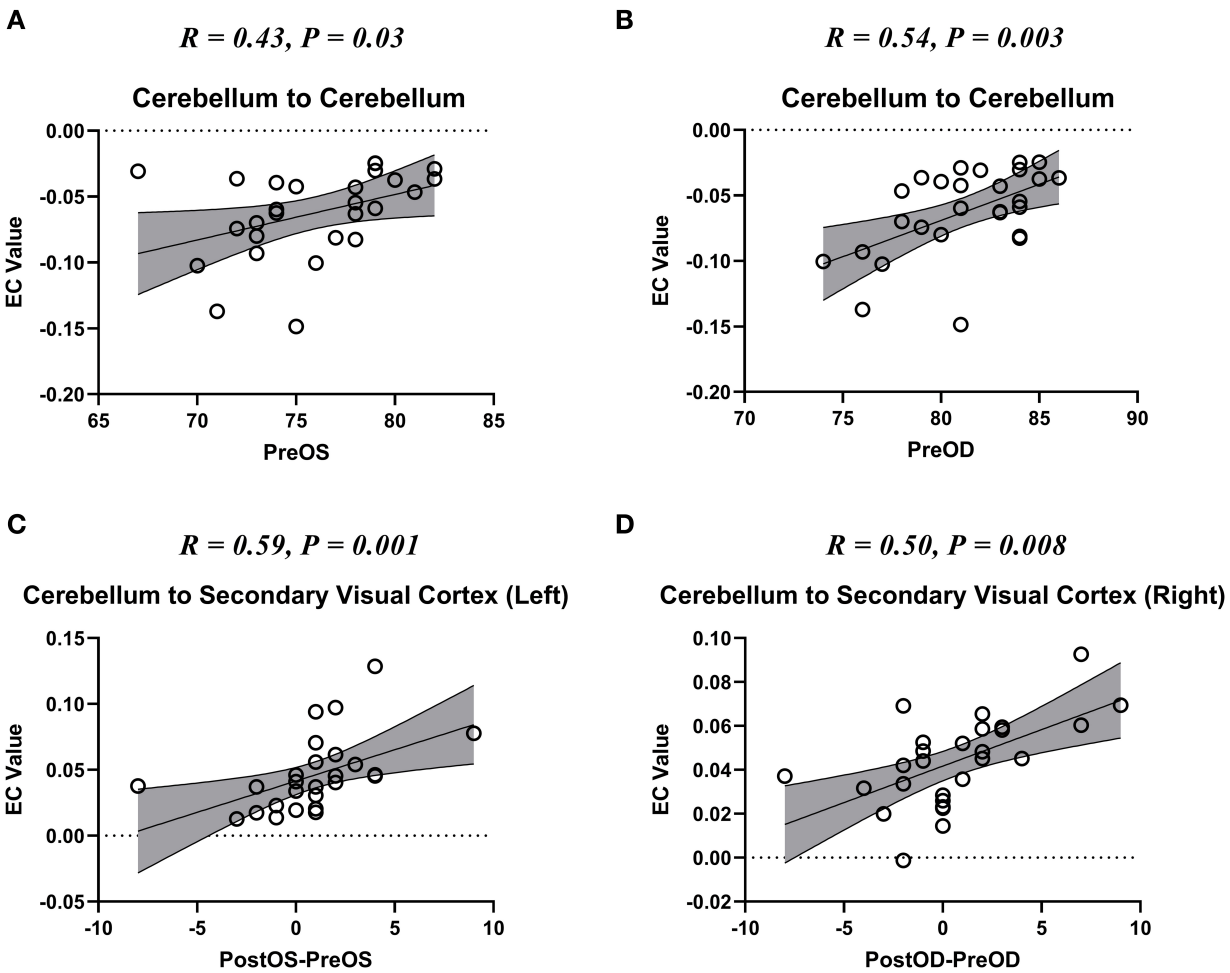
The scatter plot of the correlation between the altered effective connectivity (EC) and visual acuity. Pre-OD, pre-operative best corrected visual acuity (BCVA) of oculus dexter (OD); Pre-OS, Pre-BCVA of oculus sinister (OS); Post-OD, post-operative BCVA of OD; Post-OS, post-operative BCVA of OS. **(A,B)** The scatter plot of the correlation between the altered self-connection of cerebellum and the visual acuity if bilateral eyes. **(C,D)** The scatter plot of the correlation between the altered connection from cerebellum to bilateral secondary visual cortices and the visual acuity recovery of bilateral eyes.

We also found that the self-connection of the cerebellum showed a significant correlation with the pre-operative visual acuities of bilateral eyes (Figures 4A,B); the unidirectional connectivity from the cerebellum to left secondary visual cortex correlated with the improvement of visual acuity of the left eye after spinal cord decompression surgery (Figure 4C); and the unidirectional connectivity from the cerebellum to right secondary visual cortex correlated with the improvement of visual acuity of the right eye after spinal cord decompression surgery (Figure 4D).

### Multivariate Pattern Analysis

**Figure 5:**
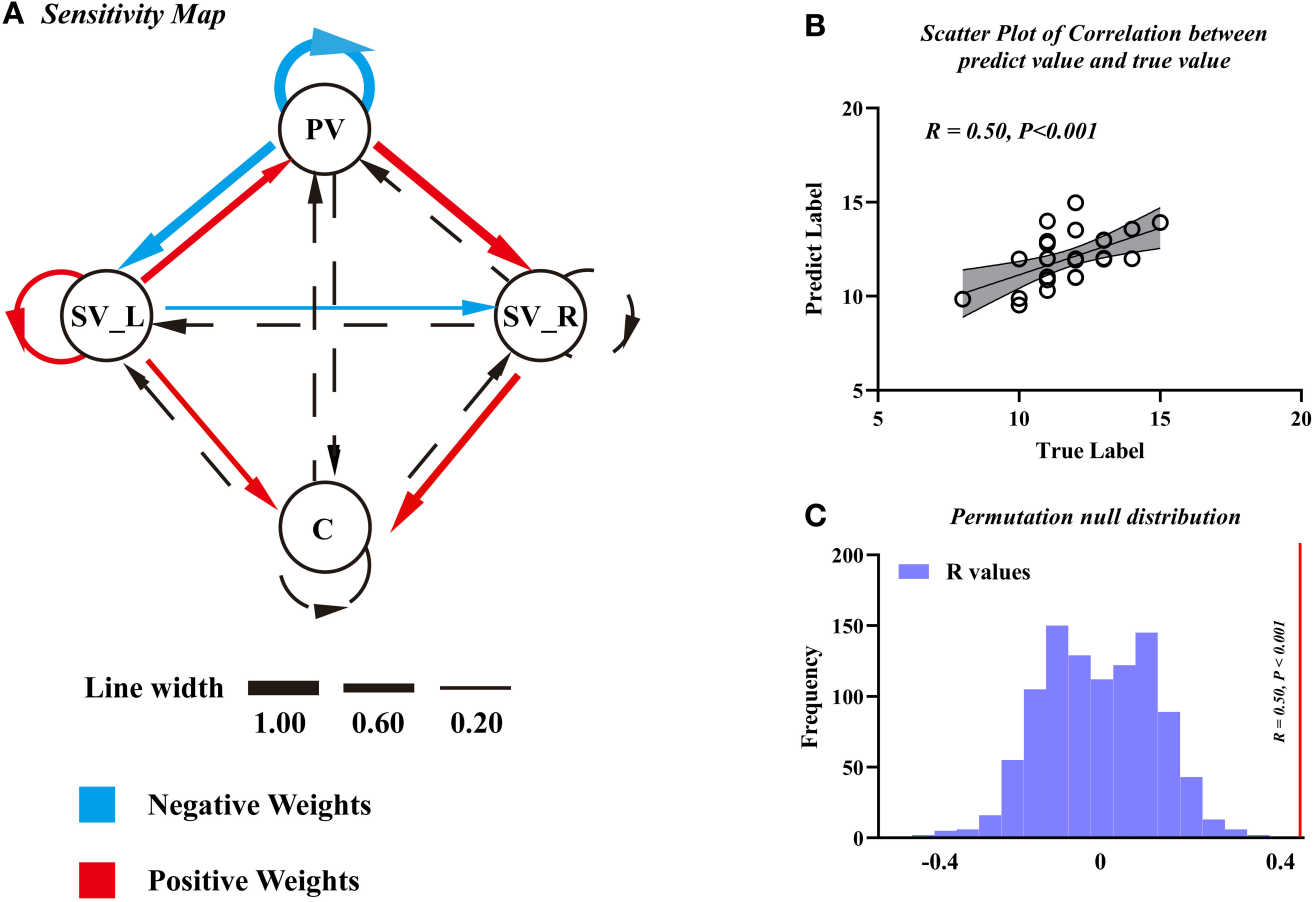
The support vector regression model of Japanese Orthopedic Association (JOA) prediction. **(A)** Sensitivity map obtained using EC as features to predict pre-operative JOA scores. **(B)** The scatter plot to predict JOA scores and the true JOA scores. **(C)** The correlation coefficient along with the corresponding null distributions. PV, primary visual cortex; SV_L, left secondary visual cortex; SV_R, right secondary visual cortex; C, cerebellum.

We found that the EC parameters among visual-cerebellum systems can be used as features to predict motor function (JOA) in patients with DCM. The final model was a seven-featured SVR model, and the selected connections are illustrated by solid lines in Figure 5A. The correlation coefficient between the predicted labels and the true labels was 0.50 (Figure 5B) and the corresponding *P*-value was < 0.001 (Figure 5C, FWE corrected); and the mean feature weight map corresponding to the prediction is shown in Figure 5A.

### Validation Analyses

To rule out the potential effects of the signal-to-noise ratio, we calculated the tSNR and compared it between two groups. We observed no significant difference of tSNR between these two groups in the ROIs defined in this study (all uncorrected *P* > 0.07, see Supplementary Figure 2).

Moreover, despite changing the cerebellum ROI (only vermis subregions) to the whole cerebellum template, our results largely remained consistent (see Supplementary Figures 3–6). Slight differences were observed in the DCM model of healthy controls, the self-connection of the cerebellum was insignificant, and the group difference of the amplitude of the cerebellum self-connection was insignificant.

We observed no significant group differences for EC among the auditory system (see Supplementary Figure 8 and Supplementary Table 2); and no significant correlation between effective connective and clinical measures was observed (see Supplementary Tables 3, 4).

## Discussion

In this study, we obtained four main results: (1) significant increases of the bidirectional connections between bilateral secondary visual cortices and cerebellum were observed in patients with DCM; (2) the self-connection of the cerebellum was positively correlated with the impaired visual acuity in patients; (3) the amplitudes of effectivity from the cerebellum to secondary visual cortices were positively correlated with better visual recovery following spinal cord decompression surgery, suggesting that patients with stronger pre-operative connectivity tend to have better outcomes following the decompression surgery in terms of visual acuity; and (4) the pattern of ECs among visual-cerebellum system could be used to predict the pre-operative JOA scores. In conclusion, these findings provided evidence that the information integration within visual cortices and cerebellum compensated for visual impairments, which might have importance for sustaining better motor function in patients with DCM.

The role of the cerebellum in modifying motor control and motor learning has been widely accepted for over decades. Nevertheless, emerging evidence has shown that it also plays a significant role in cognitive tasks and visual processing. The first discovery suggesting that cerebellum participates in visual processing was reported by Ivry and Diener. They found that the patients with cerebellar lesions were impaired in making judgments of the velocity of a moving subject, whereas elementary visual functions (i.e., visual recognition) remained intact (Ivry and Diener, 1991). Similar phenomena have also been reported. Thier and Haarmeier found that the patients with cerebellar impairments showed decreased abilities in discriminating moving subjects in the presence of visual noise (Thier et al., 1999). The above findings provide crucial evidence that the cerebellum has a potential role in the visual motion process. However, it is still unclear that how the cerebellum regulates the visual motion processing. To address this question, Baumann and Mattingley (2010) conducted their study using fMRI and illustrated that during the task, which requires participants to concentrate on the direction of a moving visual-signal input in noise, the posterior lobe of the cerebellum showed an increased activity. They concluded that the cerebellum contributes to the visual-motion detection and discrimination processes rather than the voluntary sustained allocation of attention to motion, and the cerebellum could serve a sensory support function by optimizing visual motion detection through the connections between the cerebellum and the visual, pre-frontal, and posterior parietal cortices. These results are in line with the earlier studies, in which neuronal tracers were used to examine cerebrocerebellar projections in non-human primates. It has been shown that the dorsal visual areas, which are known to participate in visual motion movement rather than visual object recognition (Perry and Fallah, 2014; Zachariou et al., 2014; Cooper and O'Sullivan, 2016; Ludwig et al., 2016; Galletti and Fattori, 2018), are anatomically connected with the cerebellum (Baumann et al., 2015). These results indicated that the cerebellum participates in the process of the visual motion information rather than other aspects of visual information. Another crucial region of regulating the visual-motion process is the LOC. It has been shown that LOC responded to consistent visual motion recognition in normal subjects, and intact lateral occipital areas are required by patients with cerebral impairments to discriminate motion direction (Zeki, 1991; Zeki et al., 1991; Tootell et al., 1995a,b; Barton et al., 1996; Huk et al., 2001; Huk and Heeger, 2002).

Considering the importance of cerebellum and LOC areas in regulating visual motion processing, in this study, the observed increased bidirectional connections between cerebellum and LOC, along with increased cerebellar self-connection (i.e., increased absolute value of the negative self-connection parameter), were crucial to understand the visual compensatory mechanism in patients with DCM. Interestingly, a similar phenomenon has also been reported. Mizelle et al. found that sensorimotor cortices, cerebellum, and LOC areas were all activated during skilled motor behavior. At the same time, reducing the visual acuity of subjects (i.e., using adjustable goggles with translucent film) caused an increase of cerebellar activation, and reducing the sensory input of subjects (i.e., inducing transient ischemic deafferentation of the distal arm) caused decreased cerebellar activation along with increased LOC activation (Mizelle et al., 2016). These findings indicated that the cerebellum and LOC are crucial parts for regulating skilled movement under visual guidance. A possible mechanism has been put forward that cerebellum and LOC simultaneously patriciate in skilled movement under intact visual and sensory inputs, and once visual and sensory inputs are reduced, the increased cerebellum and LOC function would compensate for the alterations of visual and sensory input. These results are in line with our current findings that cerebellar function increased [i.e., the more negative the self-connection we observed is, the less inhibit of the activity of this region. The detailed discussion can be found in the study by Zeidman et al. (2019a)] and associated with the visual acuity, in patients with visual impairments. It is possible that increased cerebellar function compensates for the impaired visual inputs when performing motor behavior. Besides, we also observed associations between increased connections from the cerebellum to LOC and visual recovery following the decompression surgery. These associations indicated that the increased feedback information from the cerebellum to LOC areas are important for visual recovery in patients. One possible explanation is that patients with DCM gradually adapted the altered visual input during long-term neurological dysfunctions and formed more efficient feedback modulation (from the cerebellum to bilateral LOC) to compensate for visual impairments, therefore leading to better visual recovery following surgery. These findings provided evidence that cerebellum and LOC areas participate in visual compensations, which may be important for better motor function (Chen et al., 2019) in patients with DCM. At first glance, the increased connection between the cerebellum and secondary visual cortex may seem surprising. However, a similar phenomenon (i.e., cortical plasticity and adaptive changes) has also been reported in patients with spinal cord injury (SCI). It has been shown that patients with SCI exhibited increased volume of tongue movement activation in M1 (Mikulis et al., 2002). The longitudinal task-fMRI study has also reported that in the subacute phase of SCI, increased activation volume was observed in patients with SCI than healthy controls during impaired movement. Progressive enlargement in the volume of movement-related M1 activation was also observed (Jurkiewicz et al., 2007, 2010). Moreover, increased FC and regional activities (e.g., ReHo and Alff) within sensorimotor network was also observed in the resting-state fMRI study (Min et al., 2015; Zhu et al., 2015). These functional changes have been interpreted as adaptive changes for compensating clinical symptoms (i.e., motor deficit).

To further detect the relationship between the connections and the motor function in patients with DCM, we conducted MVPA *via* the SVR method to predict the pre-operative JOA scores. In this study, we failed to detect any direct correlation (Pearson's correlation) between all EC parameters and motor function in patients. However, we found that by applying machine learning algorithm, which is more sensitive than traditional methods, after feature selection the pattern of EC parameters among visual-cerebellum systems could be used to predict the pre-operative motor function in patients with DCM. Therefore, the information process within visual cortices and cerebellum is very likely to associate with the motor behavior in patients with DCM, and this finding further supports our hypotheses that increased functional integration within the visual-cerebellum network compensates the impaired motor function by improving visual ability in patients with DCM.

In conclusion, in patients with DCM, the long-term chronic SCI could cause deafferentation leading to the symptoms of blurred vision and motor deficits. Increased EC between the cerebellum and visual cortices compensated for impaired visual ability in patients with DCM. Moreover, visual compensation might have importance for sustaining better motor function in patients with DCM.

## Limitations

This study has several limitations. First, the post-operative fMRI data were not collected due to the possible artifacts and MRI heating of implants. The fMRI data collected after spinal cord decompression were crucial for confirming the visual change in patients with DCM. The post-operative fMRI data collection requires the long-term follow-up, and we will collect them in future studies. Second, in this study, we have not selected the motor-related ROIs due to the need of reducing the complexity of the DCM model, and we will further perfect the model by adding the motor–cortex in the future to investigate the visual-motor function in patients with DCM. Third, brain diffusion tensor imaging (DTI) and diffusion spectrum imaging (DSI) data should be collected to further confirm the alteration between the visual cortex and the cerebellum in patients with DCM. Fourth, the spinal cord MR data including DTI, DSI, and functional scan should be collected in the future to further understand the neural pathophysiology mechanism of visual impairment in patients with DCM. Fifth, fMRI is known to be problematic in the posterior fossa due to distortions and susceptibility artifacts from the skull base. Despite the relatively acceptable signal-to-noise ratio in the ROIs defined in this study, future studies that conducted field correction to reduce the distortions and artifacts are needed. Finally, due to our relatively small sample size, the LOOCV performed in this study may be inadequate for validating the SVR model. We will collect more data in the future to establish a more credible model.

## Conclusion

We found that (1) significant increases of the bidirectional connections between bilateral secondary visual cortices and cerebellum were observed in patients with DCM; (2) the increased self-connection of the cerebellum was positively correlated with the impaired visual acuity in patients; (3) the amplitude of effectivity from the cerebellum to secondary visual cortices were positively correlated with better visual recovery following spinal cord decompression surgery, suggesting that patients with stronger pre-operative connectivity tend to have better outcomes following decompression surgery in terms of visual acuity; and (4) the pattern of EC among visual-cerebellum system could be used to predict the pre-operative JOA scores. In conclusion, this study provided direct evidence that visual cortices and cerebellum participate in visual compensations, which could be a crucial factor to motor deficits in patients with DCM.

## Data Availability Statement

The raw data supporting the conclusions of this article will be made available by the authors, without undue reservation.

## Ethics Statement

The studies involving human participants were reviewed and approved by Local Institutional Review Board of Tianjin Medical University General Hospital (Tianjin, China). The patients/participants provided their written informed consent to participate in this study.

## Author Contributions

YX and ML designed this study and revised this manuscript. XY, XG, HS, and XC collected the data of this study. RZ and YS performed the analysis in this study and wrote this manuscript. All authors contributed to the article and approved the submitted version.

## Conflict of Interest

The authors declare that the research was conducted in the absence of any commercial or financial relationships that could be construed as a potential conflict of interest.
