# Supplementary material for: Enhanced Information Flow From Cerebellum to Secondary Visual Cortices Leads to Better Surgery Outcome in Degenerative Cervical Myelopathy Patients: A Stochastic Dynamic Causal Modeling Study With Functional Magnetic Resonance Imaging
Source: Front Hum Neurosci. 2021 Jun 23;15:632829. doi: 10.3389/fnhum.2021.632829 (PMC8261284; doi:10.3389/fnhum.2021.632829)
Supplement: Supplementary file 1 [file Data_Sheet_1.docx]

**Sup-Figure 1.**

**
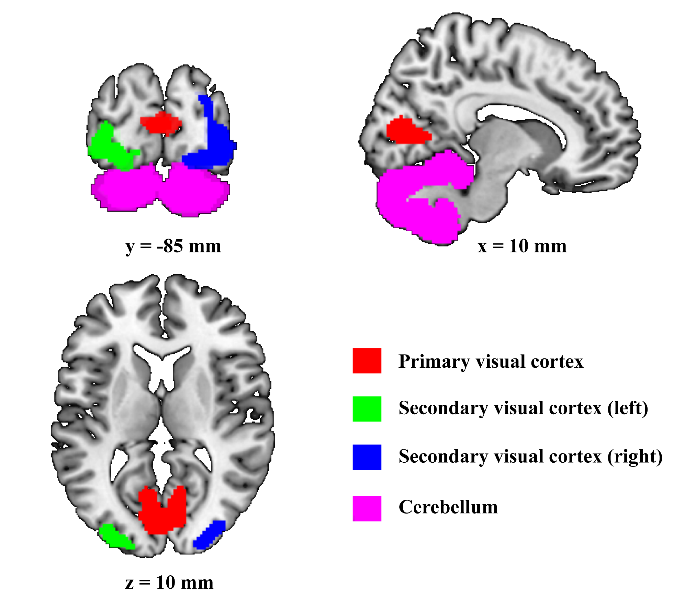
**

Location of the selected ROIs.

**Sup-Figure 2.**


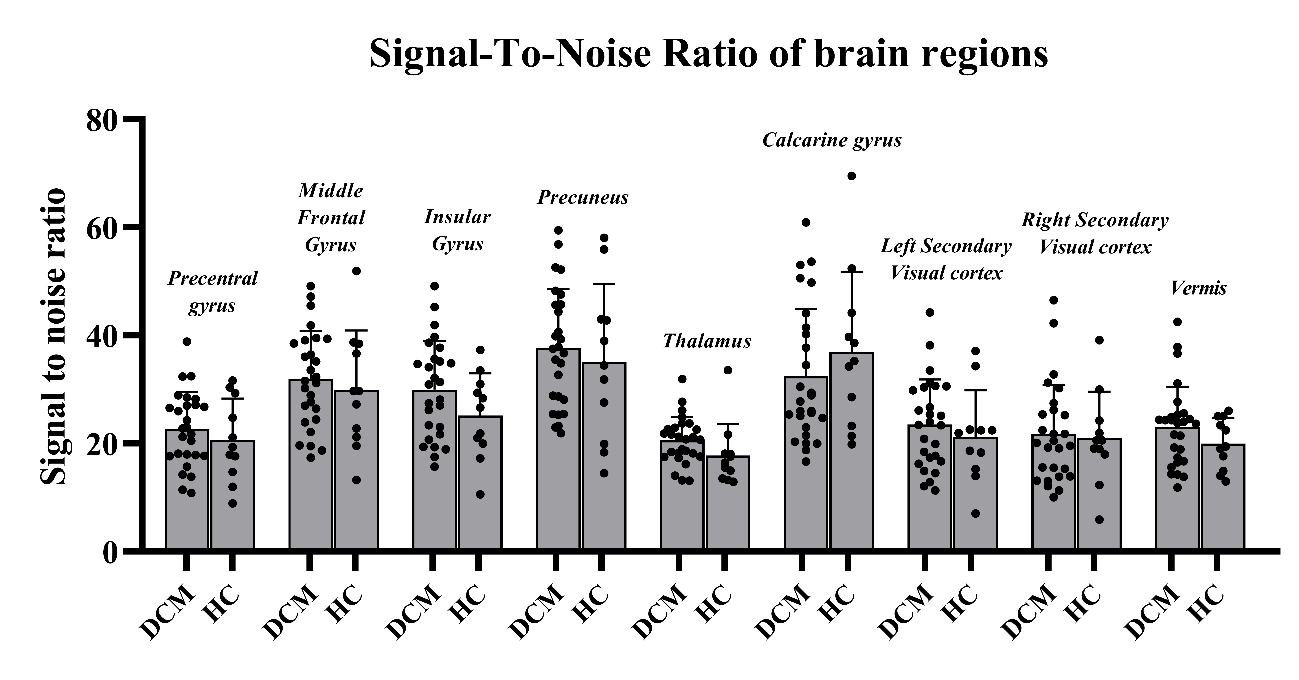


The group difference of tSNR between groups within ROIs we defined.

**Sup-Figure 3.**

**
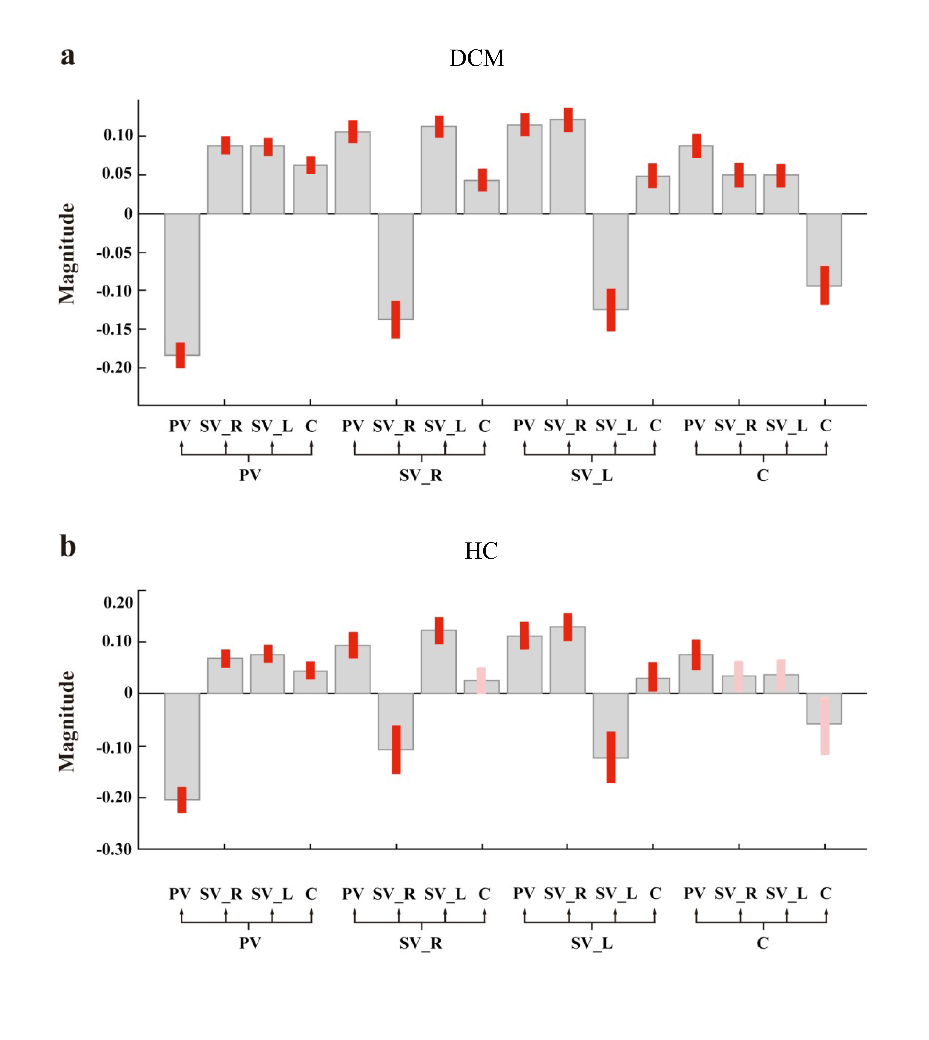
**

The group mean estimated intrinsic parameters. Grey bars represent posterior means and pink bars (or red bars) represent 95% Bayesian confidence intervals. The red bars represent the parameter whose posterior probability >0.95. PV: primary visual cortex; SV_L: left secondary visual cortex; SV_R: right secondary visual cortex; C: cerebellum.

**Sup-Figure 4.**

**
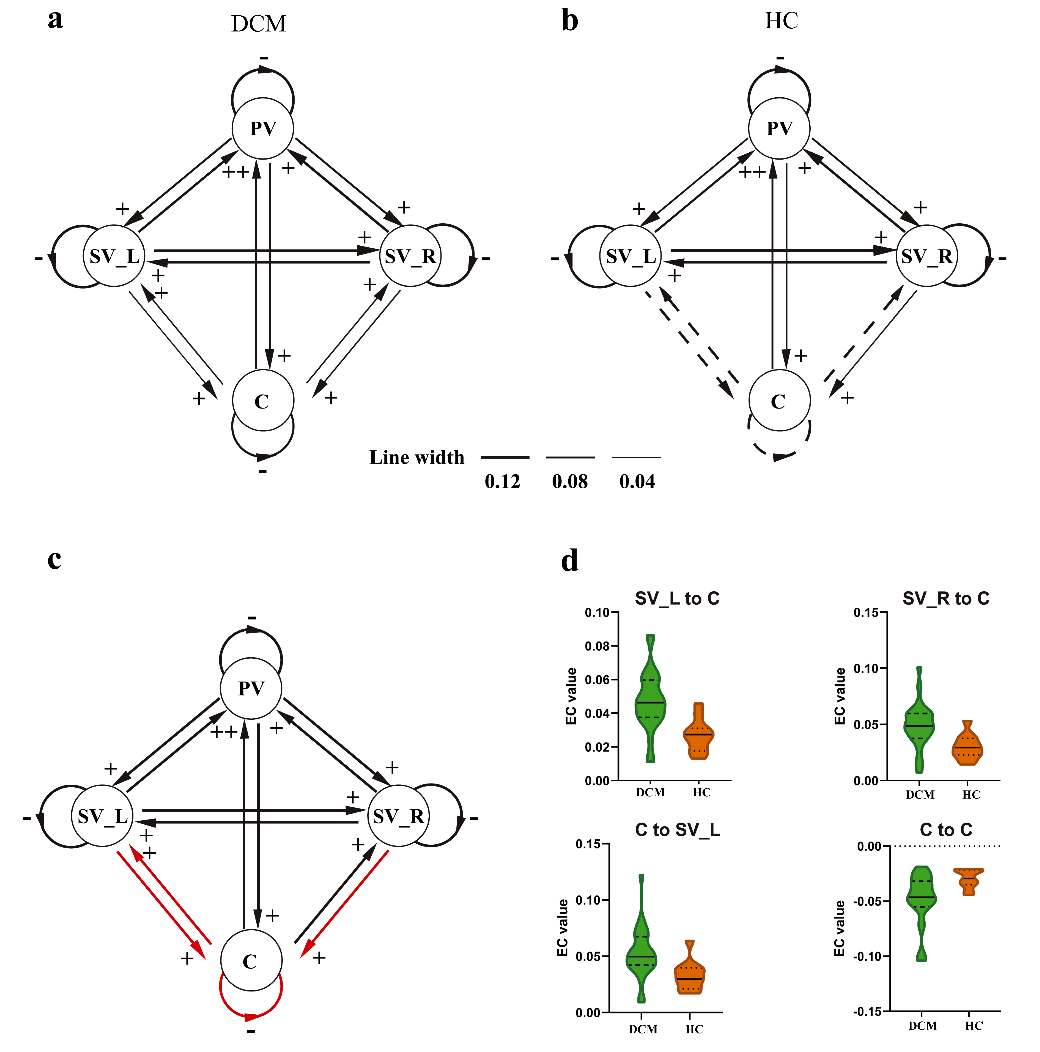
**

The hierarchical difference of visual-cerebellum network between groups. For panel. a and b; final model structure determined by PEB and BMR. Black lines with arrowhead represent the intrinsic connections in the network and the thickness of the lines represent the magnitude of each connection (the solid lines represent significant intrinsic connections and the dotted lines represent insignificant intrinsic connections). Signs beside the arrowhead indicate the sign of the intrinsic connection. Panel c. illustrated the model differences between patients and healthy controls. The red lines represent the connections showed significant differences between groups. Panel d. showed the violin plots of the connections showed significant difference between groups. PV: primary visual cortex; SV_L: left secondary visual cortex; SV_R: right secondary visual cortex; C: cerebellum.

**Sup-Figure 5.**

**
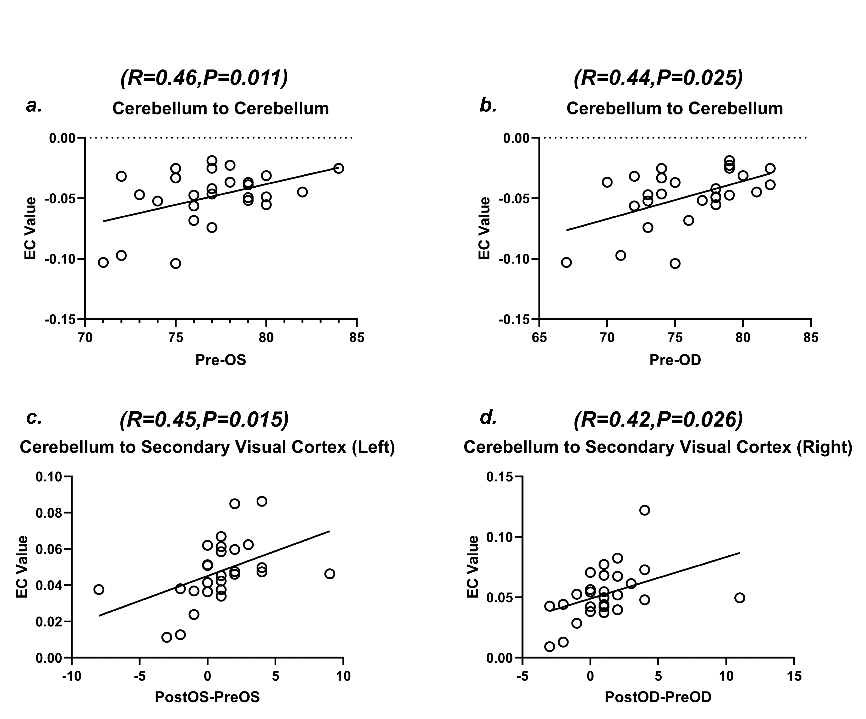
**

The scatter plot of the correlation between the altered effective connectivity and visual acuity. Pre-OD = preoperative Best Corrected Visual Acuity (BCVA) of oculus dexter (OD); Pre-OS = Pre-BCVA of oculus sinister (OS); PostOD = post-operative-BCVA of OD; PostOS = post-operative-BCVA of OS.

**Sup-Figure 6.**

**
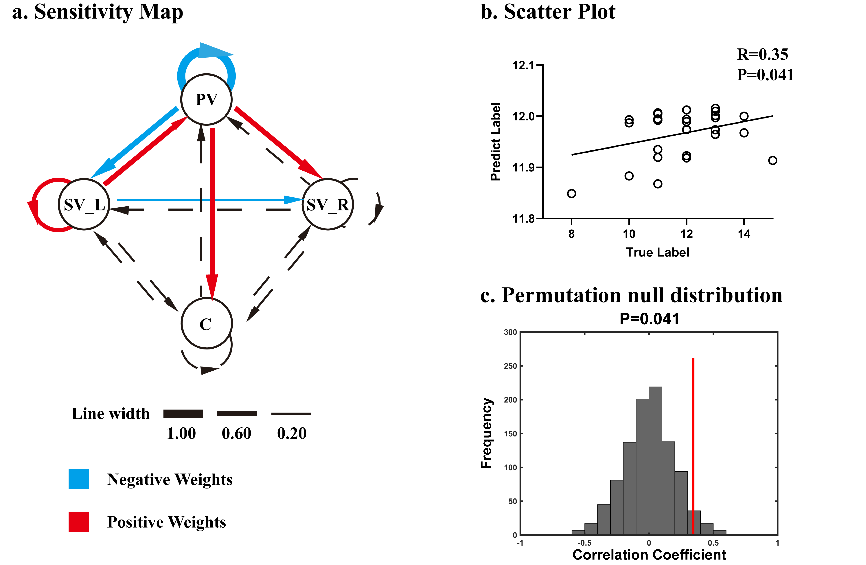
**

The Support Vector Regression model of JOA prediction. Panel a: sensitivity map obtained using effective connectivity as features to predict preoperative JOA scores. Panel b: the scatter plot of predict JOA scores and the true JOA scores. Panel c: the correlation coefficient along with the corresponding null distributions. PV: primary visual cortex; SV_L: left secondary visual cortex; SV_R: right secondary visual cortex; C: cerebellum.

**Sup-Figure 7**


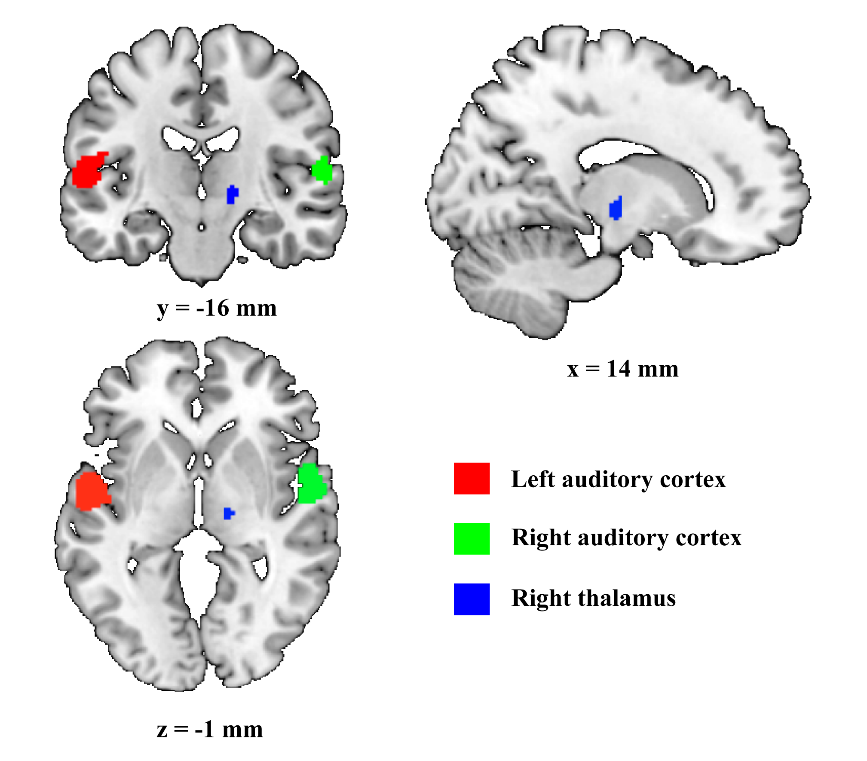


Locations of auditory ROIs used in control analysis.

**Sup-Figure 8**


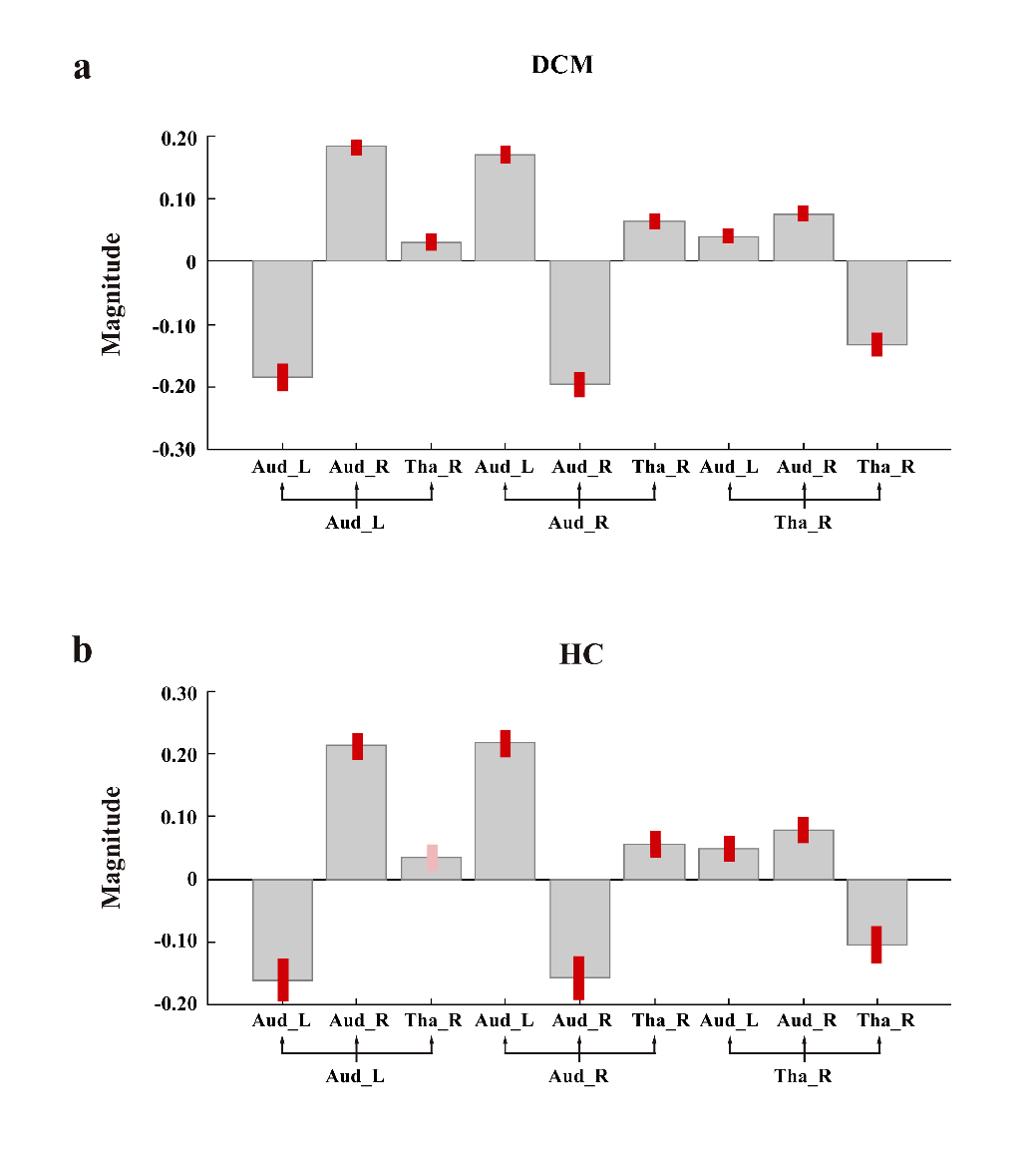


The group mean estimated intrinsic parameters. Grey bars represent posterior means and pink bars (or red bars) represent 95% Bayesian confidence intervals. The red bars represent the parameter whose posterior probability >0.95. Aud_L: left auditory cortex; Aud_R: right auditory cortex; Tha_R: right thalamus.

**Sup-Table 1.**

|  | Pre-JOA | Post-JOA | JOA-recovery |
| --- | --- | --- | --- |
| Pre_OD | **0.36*** | 0.12 | 0.13 |
| Pre_OS | **0.35*** | -0.17 | -0.02 |
| Post_OD | 0.02 | 0.21 | 0.11 |
| Post_OS | -0.08 | 0.16 | 0.17 |
| Post_OD-Pre_OD | 0.06 | 0.12 | 0.29 |
| Post_OS-Pre_OS | -0.10 | -0.07 | **0.38*** |

The correlation coefficients among clinical measures. *: p <0.05 (uncorrected). Pre: preoperative, Post: postoperative, OD: oculus dexter, OS: oculus sinister, JOA: Japanese Orthopedic Association scale

**Sup-Table 2.**

|  | ROI 1 | ROI 2 | ROI 3 |
| --- | --- | --- | --- |
| ROI 1 | 0.577 | 0.054 | 0.596 |
| ROI 2 | 0.301 | 0.061 | 0.704 |
| ROI 3 | 0.891 | 0.482 | 0.734 |

The p-value matrix of the two-sample test between degenerative cervical myelopathy (DCM) patients and healthy controls (HCs) using the effective connectivity matrix obtained from auditory system. ROI 1: left auditory; ROI 2: right auditory; ROI 3: right thalamus

**Sup-Table 3**

|  | ROI 1 | ROI 2 | ROI 3 |
| --- | --- | --- | --- |
| ROI 1 | -0.031/ 0.882 | 0.386/0.046 | -0.09/0.628 |
| ROI 2 | 0.381/0.051 | 0.096/0.632 | -0.058/0.772 |
| ROI 3 | -0.027/0.892 | -0.053/0.792 | 0.0570.776 |

The r- and p-value (uncorrected) matrix of the correlation analyses between d the effective connectivity matrix obtained from auditory system and the best corrected visual acuity (BCVA) of oculus dexter.

ROI 1: left auditory; ROI 2: right auditory; ROI 3: right thalamus

**Sup-Table 4**

|  | ROI 1 | ROI 2 | ROI 3 |
| --- | --- | --- | --- |
| ROI 1 | -0.048/0.824 | 0.341/0.065 | -0.088/0.661 |
| ROI 2 | 0.331/0.068 | 0.003/0.986 | 0.075/0.709 |
| ROI 3 | -0.085/0.671 | 0.025/0.901 | 0.058/0.771 |

The r- and p-value (uncorrected) matrix of the correlation analyses between d the effective connectivity matrix obtained from auditory system and the best corrected visual acuity (BCVA) of oculus sinister. ROI 1: left auditory; ROI 2: right auditory; ROI 3: right thalamus
